# Dual inhibitor of MDM2 and NFAT1 for experimental therapy of breast cancer: *in vitro* and *in vivo* anticancer activities and newly discovered effects on cancer metabolic pathways

**DOI:** 10.3389/fphar.2025.1531667

**Published:** 2025-02-19

**Authors:** Wei Wang, Marlene Aguilar, Sayantap Datta, Abigail Alley, Meheret Tadesse, Xinshi Wang, Xia Gao, Ruiwen Zhang

**Affiliations:** ^1^ Department of Pharmacological and Pharmaceutical Sciences, College of Pharmacy, University of Houston, Houston, TX, United States; ^2^ Drug Discovery Institute, University of Houston, Houston, TX, United States; ^3^ USDA/ARS Children’s Nutrition Research Center, Department of Pediatrics, Baylor College of Medicine, Houston, TX, United States

**Keywords:** MDM2, NFAT1, p53, triple negative breast cancer, cancer metabolism, dual inhibitor

## Abstract

**Introduction:**

The oncogene MDM2 has garnered attention not only for its role in cancer as a negative regulator of the tumor suppressor p53 but also for its p53-independent oncogenic activities. MDM2 also involves metabolic reprogramming, such as serine metabolism, respiration, mitochondrial functions, the folate cycle, and redox balance. Traditional MDM2 inhibitors blocking the protein-protein binding between MDM2 and p53 have shown limited clinical success in various stages of clinical trials, most likely due to low efficacy, drug toxicity, and drug resistance, highlighting the need for a novel, p53-independent strategy to inhibit MDM2. The present study investigated the antitumor effects of MA242, a novel MDM2 and NFAT1 inhibitor, in breast cancer models.

**Methods:**

The anticancer activity and underlying mechanisms of MA242 were evaluated in vitro using breast cancer cell lines with different p53 backgrounds and *in vivo* using orthotopic and patient-derived xenograft models.

**Results:**

We demonstrated that MA242 significantly inhibited cell viability and induced apoptosis in breast cancer cells, regardless of p53 status. Metabolic analysis revealed that MA242 notably disrupted nicotinamide metabolism, modified nucleotide metabolism, and elevated cellular oxidative stress by disturbing the redox balance. Furthermore, in animal models, MA242 reduced MDM2 expression and effectively inhibited tumor growth dependent on MDM2 expression without causing host toxicity.

**Discussion:**

These findings highlight the potential of MA242 as a modulator of cancer metabolism and support its further development as a therapeutic option for aggressive breast cancers.

## Introduction

Cancer represents a complex and multifaceted disease characterized by uncontrolled cell growth and proliferation (Hanahan and Weinberg, 2011; Hanahan, 2022). Over the past few decades, extensive research has revealed that cancer cells undergo significant metabolic alterations to sustain their rapid proliferation, establishing a critical connection between cancer biology and metabolism (Pavlova et al., 2022). Oncogenes play a critical role in reprogramming metabolic pathways to support the accelerated growth and survival of tumor cells (Martínez-Reyes and Chandel, 2021; Finley, 2023). Among these regulators, the Mouse Double Minute 2 homolog (MDM2) oncogene exerts a crucial role in regulating both tumor metabolism and cellular survival mechanisms (Rayburn et al., 2009; Wang et al., 2024).

MDM2 is a critical oncogene extensively studied for its role in carcinogenesis, cancer prevention, and treatment (Oliner et al., 2016). As an E3 ubiquitin ligase, MDM2 primarily targets the tumor suppressor protein p53 for proteasomal degradation, thereby regulating cell cycle progression, apoptosis, and genomic stability (Haupt et al., 1997; Honda et al., 1997; Kubbutat et al., 1997). Overexpression of MDM2 is common in many cancers, leading to reduced p53 function, unchecked cell proliferation, and tumor development (Oliner et al., 2016; Wang et al., 2024). This underscores the vital role of the MDM2-p53 axis in maintaining cellular homeostasis and preventing malignancy. In addition, we and others have demonstrated that MDM2 also has significant p53-independent functions that contribute to cancer development and progression (Zhang and Zhang, 2005; Bouska and Eischen, 2009; Bohlman and Manfredi, 2014). For instance, MDM2 regulates the cell cycle by modulating proteins like E2 promoter binding factor 1 (E2F1), influences apoptosis through interactions with p73, and is involved in the DNA damage response by interacting with components of the meiotic recombination 11 (MRE11)-DNA repair protein Rad50 (RAD50)-Nijmegen breakage syndrome 1 (NBS1) (MRN) complex (Eischen, 2017; Klein et al., 2021). Our lab has discovered that nuclear factor of activated T cells 1 (NFAT1), as a novel regulator of the MDM2 oncogene, directly binds to the MDM2 P2 promoter, enhancing MDM2 transcription independent of p53 (Zhang et al., 2012). Furthermore, it also promotes angiogenesis by stabilizing hypoxia-inducible factor 1α (HIF-1α), facilitating the formation of new blood vessels to supply tumors with nutrients and oxygen (Nieminen et al., 2005). Recent research has revealed that targeting the HIF-1α/transforming growth factor-β (TGF-β)/Smad signaling axis can significantly improve the immunosuppressive microenvironment and suppress breast cancer progression (Bai et al., 2025). HIF-1α is a key regulator of critical pathways such as glycolysis, angiogenesis, and metastasis, which contribute to tumor invasion, immune evasion, and drug resistance (Zhi et al., 2024). Notably, a recent study demonstrated that Ganoderic acid D overcomes gemcitabine resistance by promoting the degradation of HIF-1α driven by MDM2, which leads to a reduction in glycolysis in triple-negative breast cancer cells (Luo et al., 2024). These findings highlight the therapeutic potential of targeting the MDM2-HIF-1α axis in cancer treatment. Recently, MDM2 has garnered attention for its involvement in metabolic reprogramming. The p53 protein is particularly notable for its centrality to metabolic regulation (Labuschagne et al., 2018; Lacroix et al., 2020). Independent of p53, MDM2 significantly impacts cellular metabolism by modulating serine metabolism, respiration, mitochondrial functions, the folate cycle, and redox balance to promote tumor growth and survival (Maguire et al., 2008; Riscal et al., 2016; Arena et al., 2018; Elkholi et al., 2019).

Since its discovery, there have been extensive investigations into the discovery and development of MDM2 inhibitors for cancer therapy (Zhu et al., 2022; Li et al., 2024; Wang et al., 2024). Several MDM2 inhibitors have shown efficacy in preclinical models, and some of these MDM2 inhibitors targeting the MDM2-p53 binding have entered clinical development (Stoll et al., 2001; Vassilev et al., 2004; Ding et al., 2005; Ding et al., 2013; Wang et al., 2024). The rationale for the development of the protein-protein interaction blockers of p53-MDM2 interaction is to reduce the negative effects of MDM2 on p53 functions and protein stability, expecting the activation of p53. Of note, there is an auto-regulatory loop between p53 and MDM2: MDM2 inactivates p53, but p53 induces MDM2 expression. Preclinical cancer models have demonstrated that both p53 and MDM2 protein levels are increased in cancer cells treated with MDM2 inhibitors blocking MDM2-p53 binding. Therefore, MDM2 oncogenic effects may be increased after using such MDM2 inhibitors. Indeed, several clinical trials with these MDM2 inhibitors have not yielded successful outcomes, largely due to the stimulation of MDM2 expression resulting from p53 activation, which enhances MDM2 oncogenic activity, along with issues of drug resistance and toxicity (Wang et al., 2024). Most importantly, considering that these MDM2 inhibitors require wild-type (wt) p53 expression in cancer cells, they would be expected to have little or no activity against cancers with p53 deficiency or mutations. Unfortunately, genetic alterations of p53 are common in human cancers, and those cancers are more aggressive, more likely to metastasize, and are typically less responsive to conventional cancer therapies (Wang et al., 2024). More recently, MDM2 proteolysis-targeting chimera (PROTAC) degraders have garnered interest as potential therapeutic agents. Several MDM2-targeted PROTACs have been developed (e.g., MD-222, WB156, KT-253, MS3227, etc.), but they have only shown activity in p53 wt cells (Li et al., 2019; Wang B. et al., 2019; Yang et al., 2019; Wang et al., 2021; Marcellino et al., 2023). Recently, a newly discovered MDM2-targeted PROTAC, YX-02-030 (derived from RG7112), is the first MDM2-targeted PROTAC to demonstrate anticancer activity against p53 mutant cells in triple negative breast cancer (TNBC) (Adams et al., 2023). However, its efficacy remains limited, with a significantly higher IC_50_ (4.0–5.3 μM) (Adams et al., 2023). To our knowledge, PROTAC may cause substantial off-target toxicity and have limitations due to high molecular weight, poor solubility, and unfavorable pharmacokinetic profiles (Edmondson et al., 2019; Chen et al., 2023). Therefore, a novel, p53-independent strategy is needed to inhibit MDM2 and affirm the therapeutic value of targeting MDM2. Notably, MA242, an MDM2 inhibitor discovered in our lab, has unique mechanisms of action different from the existing MDM2 inhibitors under preclinical and clinical investigations and shows significant antitumor activity in preclinical pancreatic cancer (Wang et al., 2018) and hepatocellular carcinoma (HCC) (Wang et al., 2019b) models. MA242 directly binds to MDM2 and NFAT1 proteins with high affinity and induces their degradations. It also inhibits NFAT1-mediated MDM2 transcription by disrupting the binding of NFAT1 to MDM2’s P2 promoter (Wang et al., 2018; Wang et al., 2019b). Consequently, MA242 significantly impedes cancer cell proliferation and metastatic spread in both *in vitro* and *in vivo* models, regardless of p53 status. However, its specific effects on breast cancer, particularly TNBC, remain largely unexplored.

The overexpression and amplification of the MDM2 oncogene frequently occur in breast cancer, correlating with high tumor grade and serving as an independent negative prognostic marker in human breast cancer (Jiang et al., 1997; Turbin et al., 2006). Among breast cancer subtypes, TNBC is an aggressive subtype characterized by the absence of estrogen receptor (ER), progesterone receptor (PR), and human epidermal growth factor receptor 2 (HER2), resulting in limited therapeutic options and poorer outcomes (Bianchini et al., 2022). In a study of 214 TNBC tissues, MDM2 overexpression was observed and negatively correlated with overall survival (Park et al., 2014). In breast cancer patients with p53 mutations or deficiencies, MDM2 is still overexpressed and is linked to cancer growth, progression, poor survival, metastasis, and resistance to treatment (Jiang et al., 1997; Cuny et al., 2000; Lukas et al., 2001; Cheng et al., 2012; Zheng et al., 2023). Therefore, targeting MDM2 represents a promising strategy for developing more effective therapy for breast cancer, particularly TNBC. This study aimed to investigate the potential of MA242 as an anti-breast cancer agent by evaluating its effects on breast cancer models, especially TNBC, and its influence on cancer metabolism. By elucidating how MA242 impacts both tumor progression and metabolic pathways, this study aims to deepen the understanding of its therapeutic potential and underlying mechanisms of action.

## Materials and methods

### Chemicals, reagents, and cell lines

MA242 was synthesized and characterized in our laboratory, and the structures were confirmed by UV, IR, MS, and NMR spectroscopy. The purity of these compounds was determined to be greater than 99%. All chemicals and solvents utilized were of the highest analytical quality available. Cell culture materials and media, including phosphate-buffered saline (PBS), sodium pyruvate, non-essential amino acids, and penicillin-streptomycin, were sourced from Invitrogen (Carlsbad, CA, United States). Fetal bovine serum (FBS) was also acquired from Invitrogen. The anti-human MDM2 (Ab-2) and p21 (Ab-1) antibodies were from EMD Chemicals (Gibbstown, NJ). The anti-human p53 (DO-1) antibody was obtained from Santa Cruz Biotechnology (Santa Cruz, CA). The antibody against human NFAT1 (1/NFAT-1) was sourced from BD Biosciences (San Jose, CA), and the goat anti-mouse IgG (H + L) and goat anti-rabbit IgG (H + L) antibodies were from Bio-Rad (Hercules, CA).

Human breast cancer cells were sourced from the American Type Culture Collection (Rockville, MD, United States). All cell culture media were supplemented with 10% FBS and 1% penicillin-streptomycin. MCF7 and MDA-MB-231 cells were cultured in DMEM media.

### Analyses of cytotoxic effects

The impact of the test compound on the growth and viability of human breast cancer cells was assessed using the MTT assay, following established procedures (Wang et al., 2014a; Wang et al., 2018; Wang et al., 2019b). Cells were seeded in 96-well plates at a density of 3–4 × 10³ cells per well and exposed to the test compound at concentrations ranging from 0 to 2.5 μM for 72 h. After the incubation period, 10 μL of MTT solution (5 mg/mL; Sigma-Aldrich Co.) was added to each well, and the plates were incubated at 37°C for 2–4 h. The supernatant was then removed, and the resulting formazan crystals were dissolved in 100 μL of DMSO. Absorbance was read at 570 nm using a SYNERGY Mx microplate reader (BioTek, Winooski, VT, United States). The percentage of cell survival was determined by comparing the mean optical density (OD) of treated wells to that of the DMSO-treated control wells.

Apoptotic cell populations were detected using an Annexin V-FITC apoptosis detection kit (BioVision, Mountain View, CA, United States) according to the manufacturer’s instructions (Wang et al., 2014a; Wang et al., 2018; Wang et al., 2019b). For this analysis, 2–3 × 10⁵ cells were treated with the test compound at concentrations of 0, 0.25, 0.5, and 1 μM and incubated for 48 h. Cells were then collected, washed with serum-free media, resuspended in 500 μL of Annexin V binding buffer, and stained with 5 μL of Annexin V-FITC and 5 μL of propidium iodide. The samples were incubated in the dark at room temperature for 5 min and analyzed using a FACSCalibur flow cytometer (BD Biosciences).

To evaluate the effect of the test compound on cell cycle distribution, cells (2–3 × 10⁵ per well) were treated with the compound at concentrations of 0, 0.25, or 0.5 μM and incubated for 24 h, following standard protocols (Wang et al., 2014a; Wang et al., 2018; Wang et al., 2019b). After incubation, cells were trypsinized, washed with PBS, and fixed in 1.5 mL of 95% ethanol at 4°C overnight. The cells were then treated with RNase and stained with propidium iodide (Sigma-Aldrich Co.), followed by DNA content analysis using flow cytometry.

### Western blotting analysis

Breast cancer cells were treated with various concentrations of MA242 for 24 h. Cell lysates, each containing equal amounts of protein, were separated by SDS-PAGE and transferred to nitrocellulose membranes from Bio-Rad (Bio-Rad Laboratories, Hercules, CA, United States) following standard procedures (Wang et al., 2014a; Wang et al., 2018; Wang et al., 2019b). The membranes were blocked at room temperature for 1 h in Tris-buffered saline with 0.1% Tween 20% and 5% nonfat milk. They were then incubated overnight at 4°C with the appropriate primary antibody while being gently agitated. The next day, the membranes were washed three times for 15 min each with Tris-buffered saline containing 0.1% Tween 20. Subsequently, the membranes were incubated at room temperature for 1 h with a horseradish peroxidase-conjugated goat anti-mouse/rabbit IgG secondary antibody (Bio-Rad). After three additional washes, the target proteins were visualized using enhanced chemiluminescence reagents from PerkinElmer LAS Inc. (Boston, MA, United States).

### Immunofluorescence

MCF7 and MDA-MB-231 cells were seeded on coverslips in a 12-well plate at a density of 10,000 cells per well and allowed to adhere overnight. The cells were then treated with 0 and 0.5 μM MA242 for 24 h (Wang et al., 2014a; Wang et al., 2018; Wang et al., 2019b). After treatment, the cells were fixed, and immunofluorescent staining was conducted according to established protocols. Images were captured using a confocal microscope (Nikon Inc., Melville, NY, United States).

### Metabolite extraction

The extraction of cellular metabolites was the same as reported previously (Gao et al., 2018). MDA-MB-231 cells were treated with MA242 (0.2 μM) for 3 and 6 h, selected based on the growth curve and toxicity assays showing no significant changes in cell number or death. After treatment, the medium was removed, and cells were placed on dry ice. 1 mL of ice-cold extraction solvent (80% methanol/water) was added to each well, and the extraction plate was quenched at −80°C for 10 min. Cells were then scraped off the plate into an Eppendorf tube. Samples were vortexed and centrifuged at 20,000 x g for 10 min at 4°C. 400 μL of supernatant was transferred to a new Eppendorf tube and dried in a vacuum concentrator. The dry pellets were stored at −80°C for LC-MS analysis. Samples were reconstituted into 30 µL of sample solvent (water: methanol: acetonitrile, 2:1:1, v/v/v) and centrifuged at 20,000 x g at 4°C for 3 min. The supernatant was transferred to LC vials for analysis.

### High-performance liquid chromatography-mass spectrometry

Chromatography separations were carried out using a HILIC with an Xbridge amide column (100 × 2.1 mm internal diameter [i.d.], 3.5 µm; Waters) on the Vanquish Horizon UHPLC system. The column temperature was maintained at 40°C, with the autosampler at 4°C and the injection volume of 3 µL. The column was employed with mobile phase A: 5 mM ammonium acetate in water (pH = 9.0 adjusted with the addition of ammonium hydroxide) and mobile phase B: 100% acetonitrile. The linear gradient was: 0 min, 85% B; 1.5 min, 85% B; 5.5 min, 35% B; 10.5 min, 35% B; 10.6 min, 10% B; 14 min, 10% B; 14.5 min, 85% B, and 24 min, 85% B. The flow rate was 0.3 mL/min. The mass spectrometry analysis was performed on an Orbitrap Exploris 480 mass spectrometer equipped with a heated electrospray ionization (HESI) probe. For polar metabolites, the relevant parameters were listed: Vaporizer temperature, 120°C; sheath gas, 30; auxiliary gas, 10; sweep gas, 3; spray voltage, 3.6 kV for positive mode and 2.5 kV for negative mode. Capillary temperature was set at 320°C, and S-lens was 55. The full scan range was 60–900 (mass to charge [m/z]). The resolution was set at 240,000. Customized mass calibration was performed before data acquisition using Xcalibur.

### Mouse orthotopic and patient-derived xenograft (PDX) tumor models and animal treatment

The animal protocol received approval from the Institutional Animal Use and Care Committee of the University of Houston. Female athymic pathogen-free nude mice (nu/nu, 4–6 weeks old) were acquired from the Jackson Laboratory (Bar Harbor, ME, United States). To create MCF7 human breast cancer orthotopic models, each mouse was first implanted with a 60-day subcutaneous slow-release estrogen pellet (SE-121, 1.7 mg 17β-estradiol/pellet; Innovative Research of America, Sarasota, FL, United States). The following day, cultured MCF7 cells (5 × 10^6^ cells in a total volume of 30 µL) were transplanted into the mammary fat pad of the mice. The same procedure was followed for the MDA-MB-231 orthotopic model without the estrogen pellet. PDX models were generated by the implantation of PDX into female NOD.Cg-Prkdc^scid^ Il2rg^tm1Wjl^/SzJ (NSG) mice. In brief, patient-derived tumors were finely minced into 2 × 1 × 1 mm^3^ sections and subcutaneously transplanted into the right flanks of NSG mice while under anesthesia. The PDX tumors used in this study include TM00096 (MDM2^high^) and TM00098 (MDM2^low^) from the Jackson Laboratory. All animals were monitored for activity, physical condition, body weight, and tumor growth. Tumor size was measured every 3 days using calipers in two perpendicular diameters. Tumor volume (mm^3^) was calculated with the formula: 1/2a × b^2^, where ‘a’ is the long diameter and ‘b’ is the short diameter (in cm).

The animals with human cancer orthotopic and xenograft were randomly assigned to different treatment groups and a control group (10–15 mice per group). The untreated control group received only the vehicle. MA242 was dissolved in PEG400:ethanol:saline (57.1:14.3:28.6, v/v/v) and administered by intraperitoneal injection at a dose of 2.5 and 5 mg/kg per day (orthotopic model) or 5 mg/kg per day (PDX model) (Wang et al., 2018; Wang et al., 2019b). At the termination of the experiments, all orthotopic and xenograft tumors and other organs were excised, weighed, and snap-frozen for Western blot analysis, immunohistochemistry, and hematoxylin and eosin (H&E) staining.

### H&E staining and immunohistochemistry

Hematoxylin and eosin (H&E) staining was conducted as previously outlined (Wang et al., 2014a; Wang et al., 2018; Wang et al., 2019b). Briefly, freshly dissected tissues were fixed, embedded in paraffin, and cut into 4-μm sections. The sections were then deparaffinized and stained with Mayer’s Hematoxylin and Eosin solution. After staining, the sections were dehydrated and mounted with Permount in a fume hood. The results were examined using a phase-contrast Olympus microscope (Olympus America Inc.).

For immunohistochemical staining, freshly dissected tissues were fixed in 10% neutral buffered formalin for 24–48 h. The tissue was then embedded in paraffin, sectioned to the desired thickness with a microtome, and mounted on slides. After several wash cycles, the tumor sections were blocked and incubated with an anti-human MDM2 antibody and pre-diluted streptavidin–peroxidase horseradish peroxidase conjugates, using a staining kit from Dako North America Inc. (CA, United States). The sections were counterstained with hematoxylin and analyzed using a phase-contrast Olympus microscope (Olympus America Inc.) (Wang et al., 2014a; Wang et al., 2018; Wang et al., 2019b).

### Statistical analysis

Data analysis was performed using Prism software version 10 (GraphPad Software Inc., San Diego, CA, United States). Comparisons between the two groups were made using the Student’s t-test. Quantitative data was presented as means ± SEM from a minimum of three independent experiments. Differences were deemed statistically significant if *P* < 0.05. All statistical tests were two-sided.

The metabolite identification and peak integration were done using Thermo Scientific™ Compound Discoverer™ 3.3 software. The integrated peak intensity was used for further data analysis. Pathway analysis of metabolites was carried out with the software MetaboAnalyst 5.0 (https://www.metaboanalyst.ca/) using the KEGG pathway database (https://www.genome.jp/kegg/). All data was represented as mean ± SD unless otherwise indicated. Unless otherwise noted, the *P* values were calculated by a two-tailed Student’s t-test.

## Results

### MA242 exhibits potent cytotoxic effects in breast cancer cells

**FIGURE 1:**
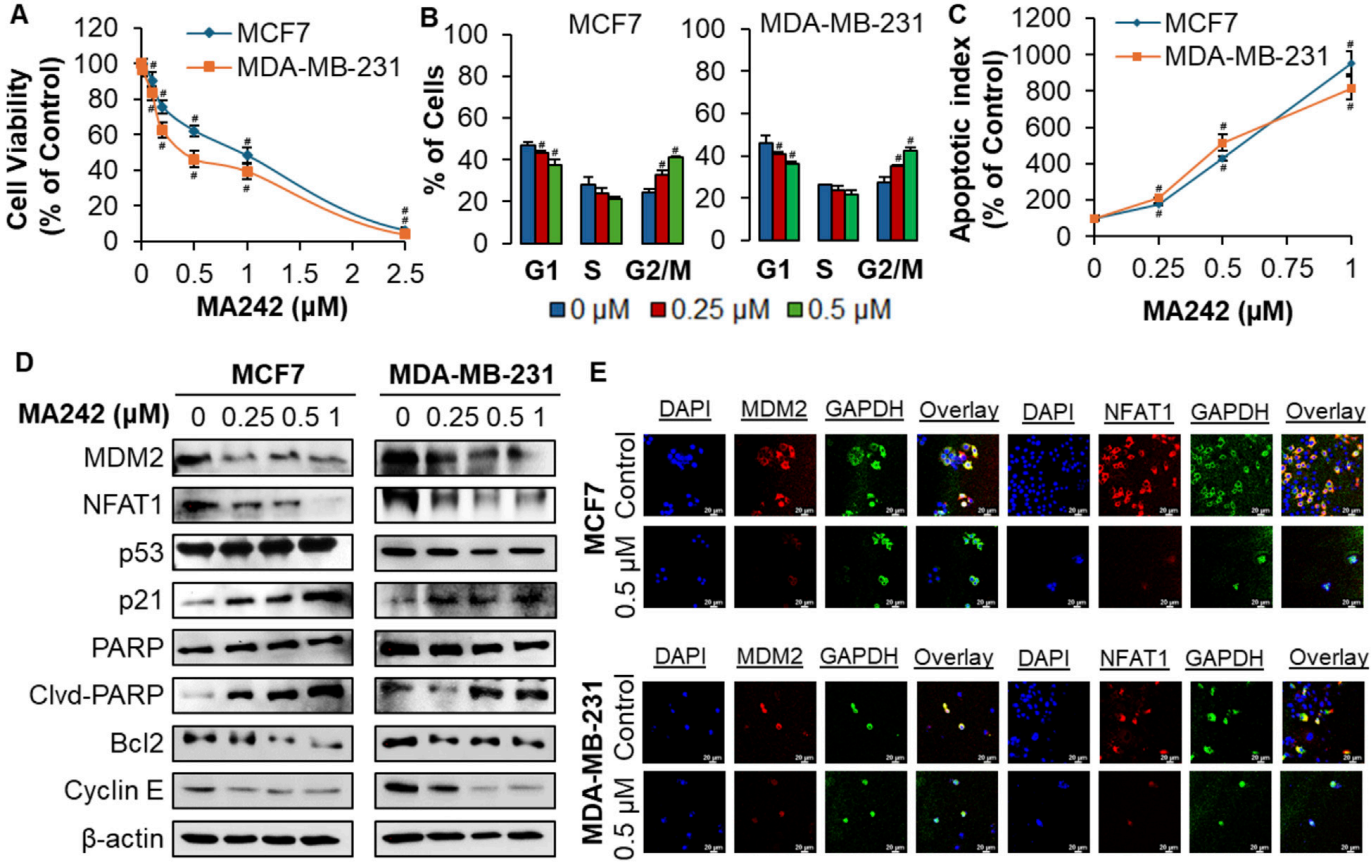
MA242 selectively inhibits breast cancer cell growth and reduces MDM2 and NFAT1 levels, independent of p53. Breast cancer cells MCF7 and MDA-MB-231 were exposed to various concentrations of MA242 for **(A)** 72 h for the MTT assay to evaluate cell viability and IC_50_ (the 50% inhibitory concentration) value; **(B)** 48 h for the apoptosis assay; **(C)** 24 h for the cell cycle distribution assay; **(D)** 24 h before Western blot analysis of the expression of MDM2 and proteins related to apoptosis and cell cycle arrest; and **(E)** representative images of MDM2 and NFAT1 immunofluorescence in control and MA242-treated human breast cancer cells. GAPDH and DAPI were used as internal controls. Quantitative data was presented as mean ± SEM, two-sided Student’s t*-*test (^#^ *P* < 0.01).

The cytotoxic impact of MA242 on breast cancer cells was assessed *in vitro* utilizing the MTT assay. The breast cancer cells were (MCF7/p53 wild type (wt) and MDA-MB-231/p53 mutant (mt)) treated with MA242 at concentrations ranging from 0 to 2.5 μM over a 72-h period, followed by evaluation of cell viability (Figure 1A). The IC_50_ values, representing the concentration required to reduce cell viability by 50%, were subsequently determined. MA242 exhibited IC_50_ values between 0.98 and 0.46 μM in MCF7 and MDA-MB-231 cells, respectively. To investigate the mechanisms by which MA242 impacts breast cancer cells, we initially examined its effects on cell cycle distribution. As shown in Figure 1B, MA242 induced a significant, concentration-dependent G2 phase cell cycle arrest in both breast cancer cell lines (*P* < 0.01). Additionally, as shown in Figure 1C, both cell lines exhibited a significant, concentration-dependent increase in apoptosis (*P* < 0.01). Specifically, treatment with 1 μM MA242 resulted in a 10-fold increase in the apoptotic index in p53 wild-type MCF7 cells and an 8-fold increase in p53 mutant MDA-MB-231 cells, compared to control cells (*P* < 0.01).

### MA242 reduces MDM2 and NFAT1 in breast cancer cells

The effect of MA242 on MDM2 expression was assessed in breast cancer cell lines. As illustrated in Figure 1D, MDM2 protein levels declined in a concentration-dependent manner in both cell lines. In MCF7 cells with wild-type p53, there was an increase in p53 protein levels, likely due to MDM2 inhibition. Mechanistically, our previous research on pancreatic cancer (Wang et al., 2018) and hepatocellular carcinoma (HCC) (Wang et al., 2019c) demonstrated that MA242 destabilizes the MDM2 protein by shortening its half-life and promoting its degradation through the proteasome. This mechanism effectively prevents MDM2-mediated degradation of wild-type p53, leading to its accumulation (Wang et al., 2018; Wang et al., 2019c). Moreover, the expression of p21Waf1/CIP1, a gene regulated by MDM2, was elevated in both cell lines, further indicating MDM2 inhibition. Additionally, MA242 influenced the expression of various proteins related to apoptosis and the cell cycle: it elevated the levels of cleaved PARP while reducing the levels of Bcl-2 and Cyclin E, pointing to a p53-independent mechanism. The downregulation of MDM2 and NFAT1 by MA242 was further validated through immunofluorescence analysis. Compared to the control cells, MA242-treated cells exhibited a significant reduction in MDM2 and NFAT1 expression in both cell lines (Figure 1E), suggesting that MA242 effectively targets and suppresses these proteins at the cellular level, highlighting its promise as a potential therapeutic agent for breast cancer.

### MA242 demonstrates *in vivo* antitumor activity in orthotopic models

**FIGURE 2:**
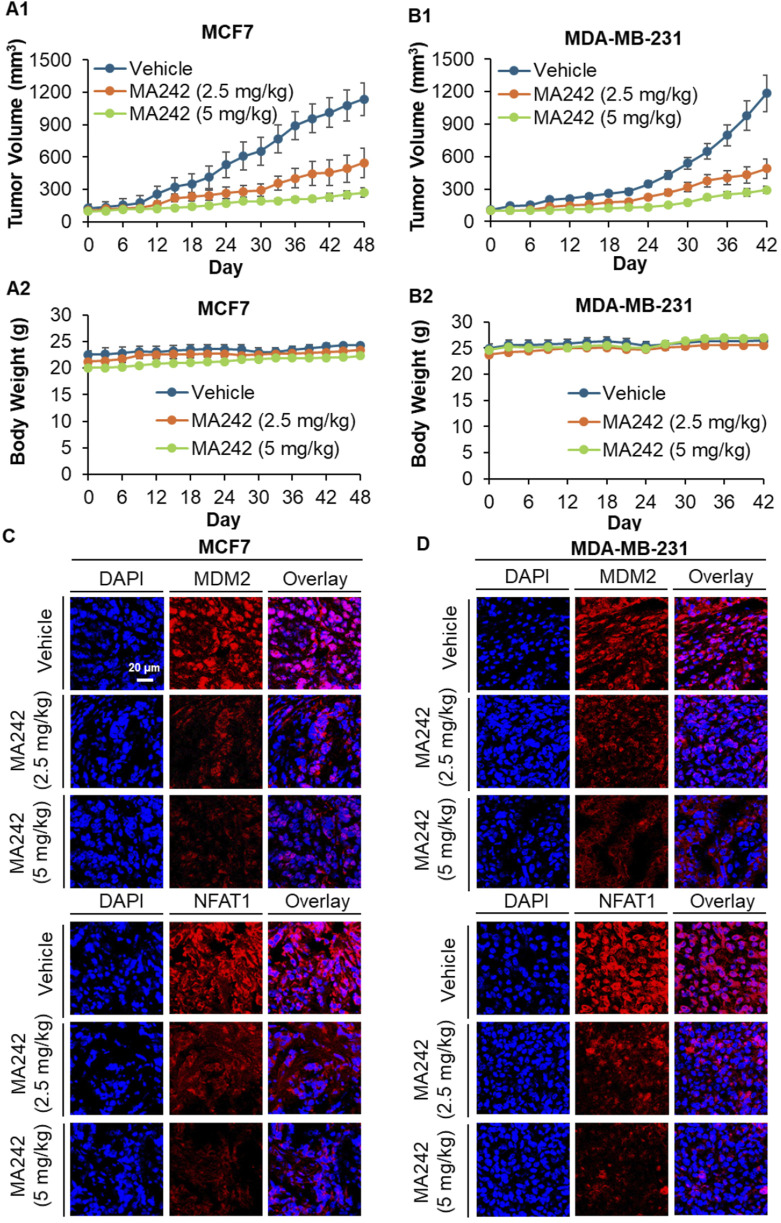
MA242 inhibits orthotopic breast cancer growth and decreases MDM2 and NFAT1 expression, independent of p53. **(A1)** MCF7 and **(B1)** MDA-MB-231 cells were implanted orthotopically into the mammary fat pads of nude mice. Mice were treated with MA242 by i.p. injection at 2.5 and 5 mg/kg/d, 5 d/wk for 48 and 42 days in the MCF7 and MDA-MB-231 models, respectively (^#^ *P* < 0.01). **(A2, B2)** The mice were monitored for changes in body weight as a surrogate marker for toxicity. At the end of the experiments, the orthotopic tumors were carefully removed and analyzed via immunohistochemical staining in the **(C)** MCF7 and **(D)** MDA-MB-231 models (all images represent serial sections; scale bar, 20 μm).

*In vivo* efficacy of MA242 in breast cancer MCF7 and MDA-MB-231 orthotopic tumor models was investigated next. Nude mice bearing MCF7 and MDA-MB-231 orthotopic tumors received treatment with or without MA242 (2.5 and 5 mg/kg/day, 5 days/week) via intraperitoneal injection for 48 and 42 days, respectively. The dosage selection for breast cancer treatment was based on initial Maximum Tolerated Dose (MTD) and safety studies. MA242 treatment resulted in a significant inhibition of tumor growth by 54.2% and 76.7% in the MCF7 orthotopic model (*P* < 0.01, Figure 2A1) and 59.5% and 74.6% in the MDA-MB-231 orthotopic model (*P* < 0.01, Figure 2B1), respectively, compared to control mice. Notably, there were no significant changes in the average body weights of either control or MA242-treated mice, indicating minimal host toxicity induced by MA242 (Figures 2A2, B2). Immunohistochemistry analysis of all tumors revealed decreased expression levels of both NFAT1 and MDM2 in MA242-treated tumors (Figures 2C, D).

### MA242 inhibits tumor growth in TNBC PDX models in an MDM2 expression-dependent manner

**FIGURE 3:**
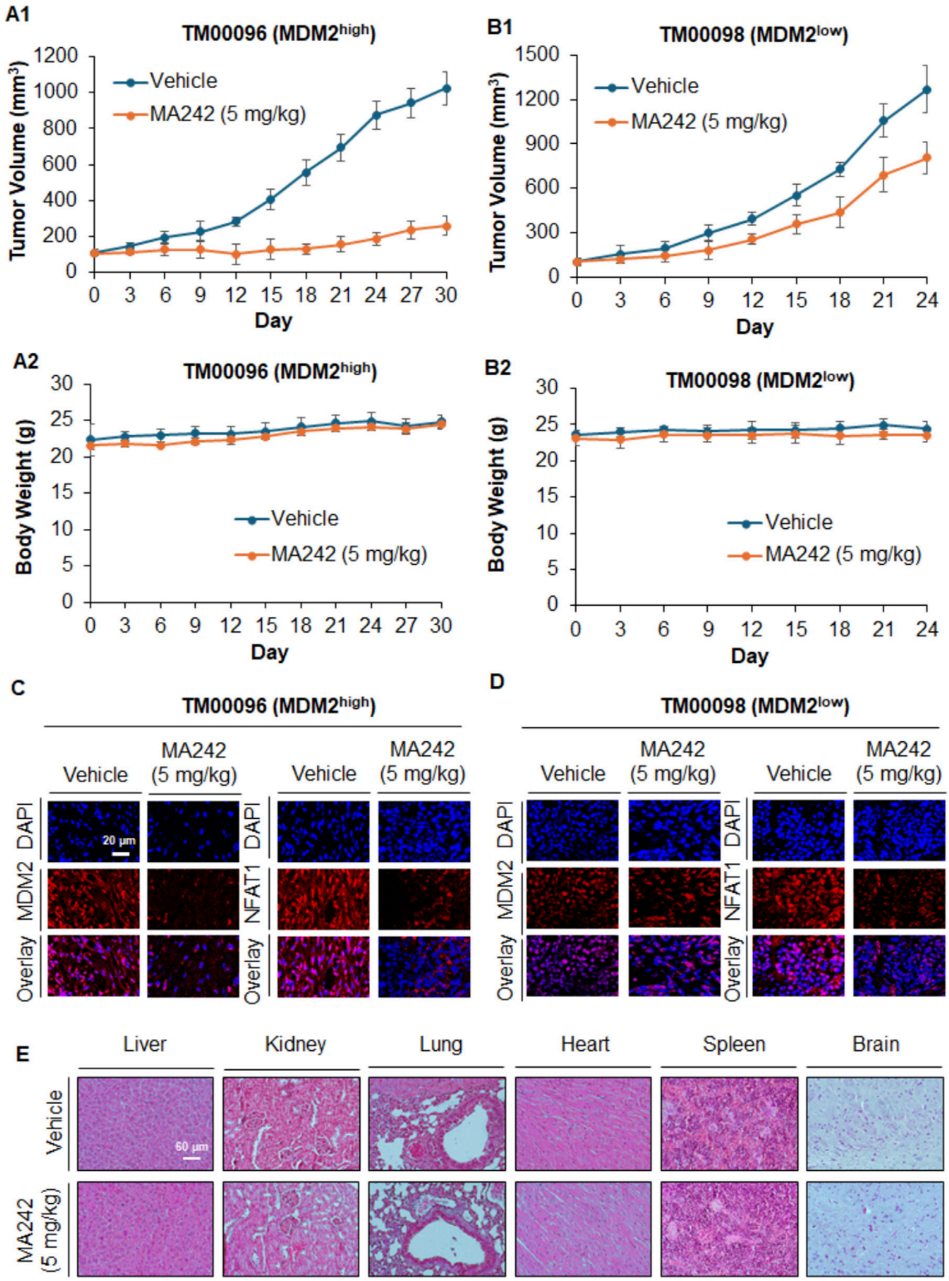
MA242 inhibits orthotopic PDX breast cancer growth, depending on the MDM2 expression level and demonstrating its targeting specificity. TM00096 (MDM2^high^) and TM00098 (MDM2^low^) tissues from patients were implanted into the right flanks of female NSG mice. MA242 was administered via i.p. injection at 5 mg/kg/d or 5 d/wk for 30 or 24 days, respectively. Tumor growth curves of **(A1)** TM00096 (MDM2^high^) and **(B1)** TM00098 (MDM2^low^) xenograft tumors are shown (^#^ *P* < 0.01). **(A2, B2)** The mice were monitored for changes in body weight as a surrogate marker for toxicity. **(C, D)** Upon termination of the experiments, the tumors were removed, and the protein expression of MDM2 and NFAT1 was analyzed by immunohistochemistry (scale bar, 20 μm). **(E)** At the termination of the experiments, H&E staining of paraffin sections of major organs from mice bearing TM00098 tumors was performed (scale bar, 60 μm).

PDX models are widely recognized in translational cancer research for their ability to accurately mimic the genetic and histological features of human tumors. Compared to traditional models, PDX systems provide more reliable predictions of clinical outcomes, making them essential tools for evaluating the efficacy of novel cancer therapies (Liu et al., 2023). In this study, we investigated the effectiveness of MA242 against TNBC using PDX models. Immunodeficient NSG mice were used to establish the TNBC PDX tumors. The tumors were categorized based on their MDM2 expression levels: high MDM2 expression (TM00096) or low MDM2 expression (TM00098). MA242 was administered, and its impact on tumor growth was closely monitored. As shown in Figure 3A1, MA242 significantly inhibited the growth of TM00096 xenograft tumors, reducing their size by approximately 75.02% on Day 30 (*P* < 0.01). In contrast, MA242 had minimal impact on TM00098 xenograft tumors, which exhibited low MDM2 expression. These tumors showed no significant difference in growth compared to untreated controls (Figure 3B1). These results highlight the dependence of MA242's therapeutic efficacy on MDM2 expression levels, emphasizing its specificity for targeting tumors with high MDM2 expression.

In addition to evaluating tumor growth, we also monitored body weights to assess the potential toxicity of MA242. No significant differences in body weight were observed across the treatment groups (Figures 3A2, B2), suggesting that MA242 was well-tolerated and did not cause noticeable toxicity at its effective dose. To further understand the mechanism underlying MA242's antitumor effects, we analyzed MDM2 levels in PDX tumors using immunohistochemistry. As expected, MA242 treatment resulted in a marked reduction in MDM2 levels in TM00096 tumors, which are characterized by high MDM2 expression (Figure 3C). This significant reduction in MDM2 is consistent with the observed inhibition of tumor growth and highlights the importance of MDM2 downregulation in the anticancer activity of MA242. Conversely, in the TM00098 model, which exhibits low MDM2 expression, MA242 treatment did not significantly alter MDM2 levels (Figure 3D). This finding further emphasizes the critical role of MDM2 in the therapeutic action of MA242. Histological examinations of the treatment group with TM00098 tumors revealed no gross abnormalities in major organs, including the liver, lung, kidney, spleen, heart, or brain (Figure 3E). The same results were obtained in TM00096 model and breast cancer MCF7 and MDA-MB-231 orthotopic model (data not shown).

### MA242 impacts cancer metabolism

**FIGURE 4:**
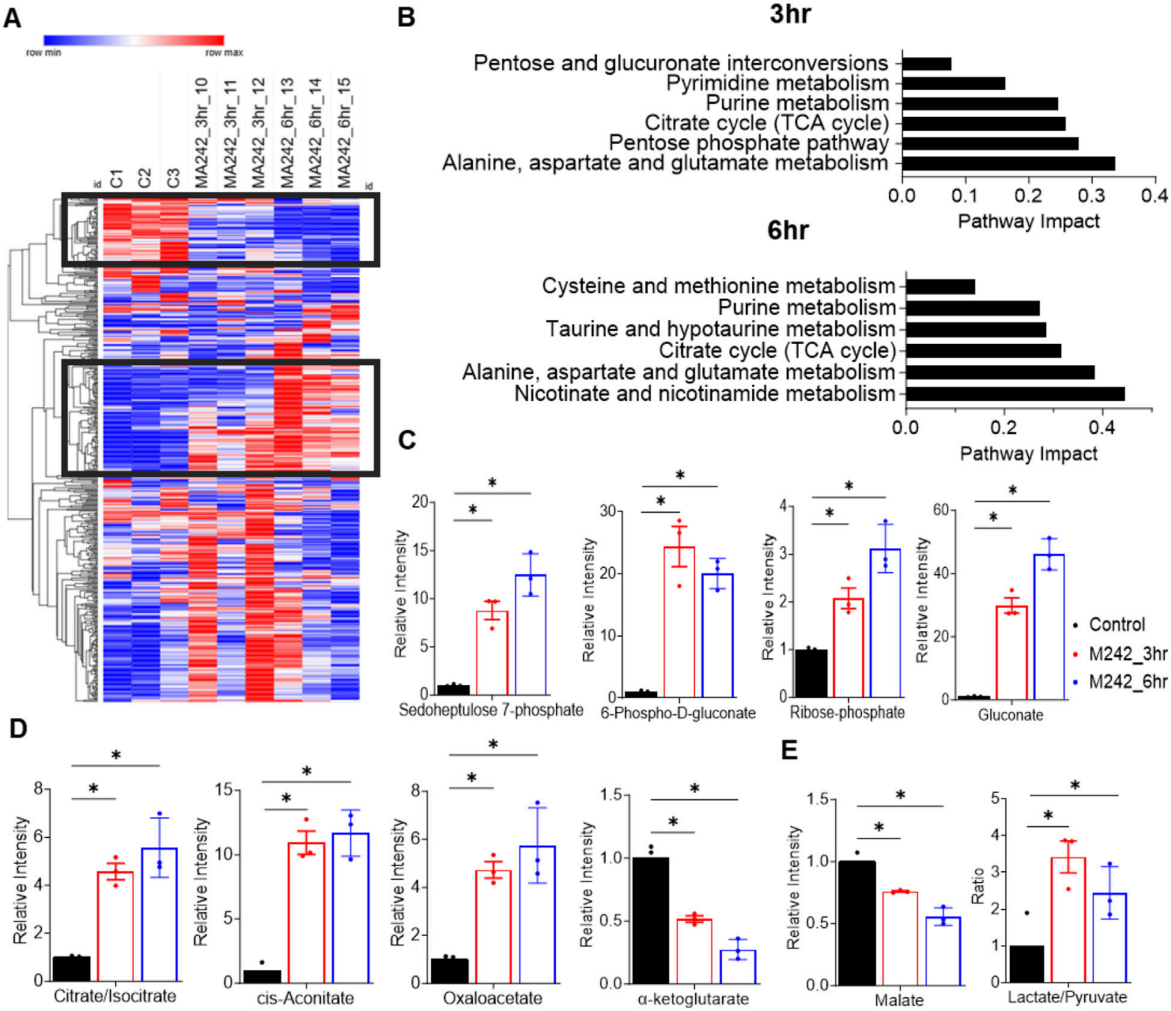
MA242 treatment dramatically impacts the pentose phosphate pathway and the TCA cycle. **(A)** Heatmap of all metabolites detected in MDA-MB-231 cells treated with vehicle (DMSO) or MA242 for 3 h or 6 h. **(B)** Pathway analysis of significantly changed metabolites (*p < 0.05 and |log2FC|>1) after 3 h or 6 h of MA242 treatment. **(C)** LC-MS measurement of intracellular sedoheptulose 7-phosphate, 6-phospho-D-gluconate, ribose 5-phosphate, and gluconate within the pentose phosphate pathway. **(D)** Quantification of TCA cycle intermediates, including citrate/isocitrate, cis-aconitate, oxaloacetate, and α-ketoglutarate. **(E)** Measurement of the additional TCA cycle intermediate malate and the lactate/pyruvate ratio. n = 3 independent samples. The means ± SDs are presented. *P* values are derived from a two-tailed, unpaired t-test (*P < 0.05).

To investigate the metabolic impact of MA242, we performed metabolomics analysis on cells treated with vehicle DMSO or MA242 at 0.2 µM for 3 or 6 h. MA242 induced significant metabolic alterations in a time-dependent manner (Figure 4). To assess the overall metabolic impact, we performed pathway analysis using metabolites that exhibited significant changes with *P* < 0.05 and |log_2_FC|>1 compared to the control group. After 3 h of treatment, the most impacted pathways, revealed by 40 significantly changed metabolites, included amino acid metabolism (alanine, aspartate, and glutamate), the pentose phosphate pathway, the TCA cycle, purine metabolism, and pyrimidine metabolism (Figure 4A). By 6 h of treatment, MA242 additionally affected nicotinamide metabolism, taurine and homotaurine metabolism, and cysteine and methionine metabolism (Figure 4B).

#### Stimulation of the pentose phosphate pathway

MA242 dramatically elevated the levels of sedoheptulose 7-phosphate, 6-phospho-D-gluconate, ribose-5-phosphate, and gluconate, indicating a strong stimulation of the pentose phosphate pathway. This pathway is crucial for producing NADPH and ribose-5-phosphate, supporting antioxidant defenses and nucleotide synthesis (Figure 4C).

#### Modulation of the TCA Cycle

Within the TCA cycle, MA242 significantly elevated the levels of citrate/isocitrate, cis-aconitate, and oxaloacetate and reduced the levels of α-ketoglutarate and malate (Figures 4D, E). These changes suggest enhanced flux through the initial steps of the TCA cycle and alterations in the balance of catabolic and anabolic processes, reflecting MA242's impact on central carbon metabolism. In addition, there was a noticeable trend showing increased levels of pyruvate and lactate, along with a significant shift in their ratio (Figure 4E). This change suggests a possible alteration in metabolic activity, likely due to disruptions in essential biochemical pathways. It may reflect adjustments in cellular respiration or energy metabolism.

#### Alterations in nucleotide metabolism

**FIGURE 5:**
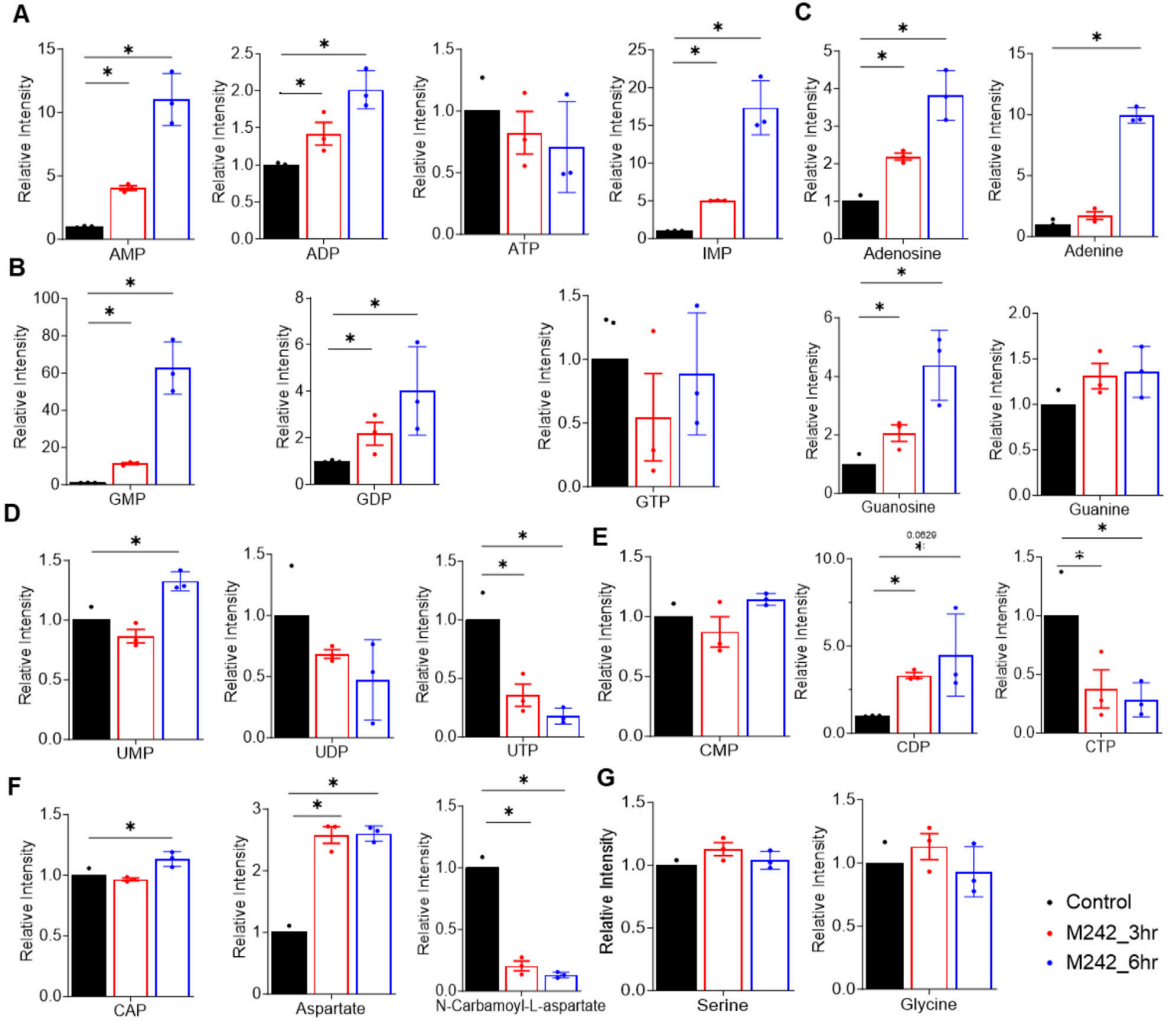
MA242 modulates nucleotide metabolism. Metabolites were detected in MDA-MB-231 cells treated with vehicle (DMSO) or MA242 for 3 h or 6 h **(A)** LC-MS quantification of AMP, ADP, ATP, and IMP. **(B)** Measurement of GMP, GDP, and GTP levels. **(C)** Analysis of adenosine, adenine, guanosine, and guanine in the purine metabolism pathway. **(D)** Quantification of UMP, UDP, and UTP. **(E)** Evaluation of CMP, CDP, and CTP levels. **(F)** Measurements of CAP, aspartate, and N-carbamoyl-L-aspartate. **(G)** Quantification of serine and glycine. n = 3 independent samples. The means ± SDs are presented. *P* values are derived from a two-tailed, unpaired t-test (*P < 0.05). ADP, adenosine diphosphate; AMP: adenosine monophosphate; ATP, adenosine triphosphate; CAP, carbamoyl phosphate; CDP, cytidine 5’-diphosphate; CTP, cytidine triphosphate; GDP, guanosine diphosphate; GMP: guanosine monophosphate; GTP, guanosine triphosphate; IMP, inosine monophosphate; UMP, uridine 5'-monophosphate; UTP, uridine triphosphate.

In addition, the levels of adenosine and guanosine nucleotides, AMP, ADP, GMP, and GDP were elevated in a time-dependent manner following MA242 treatment with no significant change in ATP or GTP (Figures 5A, B). Consistently, adenine, adenosine, guanosine, and inosine monophosphate (IMP), a purine precursor, were all increased time-dependently by MA242 treatment (Figure 5C), suggesting MA242 stimulates purine synthesis. Moreover, MA242 affected pyrimidine metabolism. Specifically, UMP and CDP levels increased, whereas UTP and CTP levels decreased (Figures 5D, E). The observed reduction in carbamoyl-aspartate, combined with elevated aspartate levels, indicates an impairment in *de novo* pyrimidine synthesis (Figure 5F). The alterations in purine and pyrimidine metabolism are unlikely to result from a shortage of one-carbon units, as levels of serine and glycine, key donors in one-carbon metabolism, remained unchanged (Figure 5G). This suggests that the metabolic changes induced by MA242 are more specific to nucleotide metabolism pathways rather than a general shortage of methyl donors. Notably, alterations in L-aspartate, N6-(1,2-dicarboxyethyl)-AMP, citrate, oxaloacetate, and N-carbamoyl-L-aspartate levels are directly related to both the TCA cycle and nucleotide metabolism. These shifts highlight the broader metabolic impact of MA242, suggesting that it may influence cellular energy balance, biosynthesis, and regulatory mechanisms within central carbon metabolism.

#### Impact on cysteine and methionine metabolism

**FIGURE 6:**
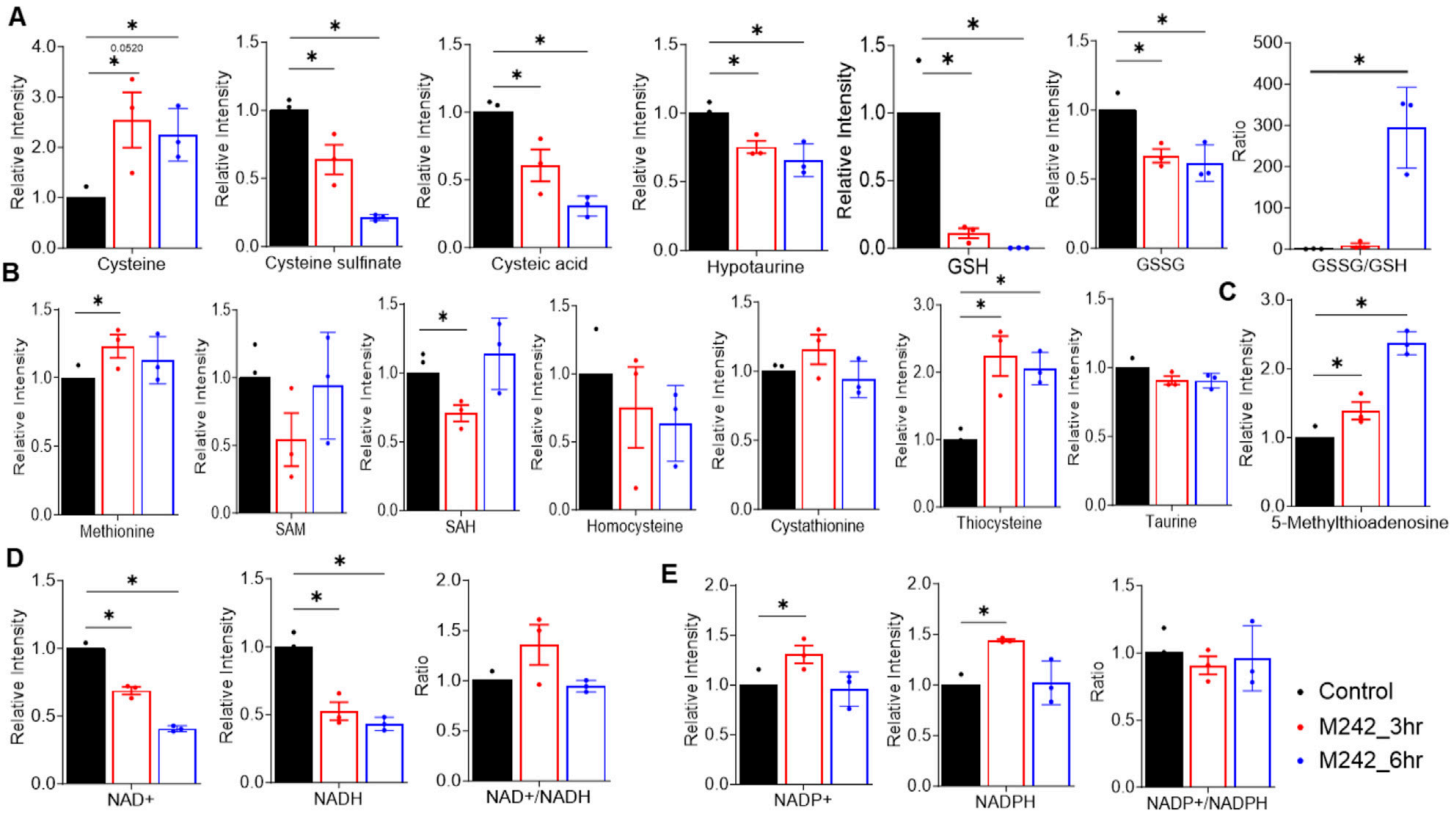
MA242 disrupts nicotinamide, cysteine, and methionine metabolism. Metabolites were detected in MDA-MB-231 cells treated with vehicle (DMSO) or MA242 for 3 h or 6 h. **(A)** Analysis of metabolites in the transsulfuration pathway, including cysteine, cysteine sulfinate, cysteic acid, hypotaurine, GSH, GSSG, and the GSSG/GSH ratio. **(B)** Quantification of methionine, SAM, SAH, homocysteine, cystathionine, thiocysteine, and taurine. **(C)** Measurement of 5-methyladenosine in the methionine salvage pathway. **(D)** Quantification of NAD^+^ and NADH levels and the NAD^+^/NADH ratio. **(E)** NADP^+^ and NADPH levels and the NADP^+^/NADH ratio in the nicotinamide metabolism pathway were measured. n = 3 independent samples. The means ± SDs are presented. *P* values are derived from a two-tailed, unpaired t-test (*P < 0.05). SAH: S-adenosylhomocysteine; SAM, S-adenosylmethionine; GSH, glutathione; GSSG, oxidized glutathione.

MA242 dramatically influenced cysteine and methionine metabolism, particularly by impairing the transsulfuration pathway (Figures 6A, B). While the core metabolites in the methionine cycle—methionine, S-adenosylmethionine (SAM), S-adenosylhomocysteine (SAH), and homocysteine—remained unchanged, cysteine levels more than doubled (Figures 6A, B). However, the downstream metabolites of cysteine, including cysteine sulfinate, cysteic acid, hypotaurine, glutathione (GSH), and oxidized glutathione (GSSG), were significantly reduced. This showed a notable increase in the GSSG/GSH ratio (Figure 6A), indicating that MA242 induces oxidative stress. The link between cysteine metabolism and redox regulation is primarily mediated through the GSH/GSSG system, underscoring the importance of cysteine and methionine metabolism in cellular redox balance. Additionally, MA242 increased the level of 5-methyladenosine in the methionine salvage pathway, which releases adenine, consistent with the observed changes in purine metabolism (Figure 6C).

#### Disruption of nicotinamide metabolism

Alongside its effects on cysteine and methionine metabolism, MA242 also disrupted nicotinamide metabolism. After 6 h of treatment, MA242 significantly reduced the levels of NAD^+^ and NADH, though the NAD^+^/NADH ratio remained unchanged (Figure 6D). This reduction in NAD^+^ and NADH aligned with observed changes in the TCA cycle. Interestingly, the levels of NADP^+^ and NADPH were not significantly affected by MA242 (Figure 6E), suggesting selective modulation of the nicotinamide adenine dinucleotide pools.

Together, MA242 treatment significantly altered nucleotide metabolism, impaired nicotinamide metabolism, and increased cellular oxidative stress by affecting the redox balance. These comprehensive metabolic changes induced by MA242 highlight its broad impact on cellular metabolic pathways, which may have important implications for its biological and therapeutic effects.

## Discussion

In the present study, we provided comprehensive insights into the potent anticancer properties of MA242 in both *in vitro* and *in vivo* breast cancer models, especially in TNBC models. Our findings emphasized MA242’s inhibitory effects on MDM2 and NFAT1, as well as its potential impact on cancer cell metabolism. MA242 demonstrated a significant cytotoxic effect across various breast cancer cell lines, presenting a compelling case for its potential as a therapeutic agent in cancer treatment.

The low IC_50_ values of MA242 demonstrated its strong ability to suppress breast cancer cell viability, even at minimal concentrations, regardless of p53 status. This underscored its potential as an effective therapeutic agent for various breast cancer subtypes. The significant increases in apoptosis and G2 phase cell cycle arrest observed in breast cancer cells with different p53 genetic backgrounds highlighted MA242's broad-spectrum efficacy. This characteristic was particularly valuable for treating p53-mutant cancers, which often show resistance to standard therapies.

The marked reduction in MDM2 levels following treatment with MA242 emphasized its targeted mechanism of action. By inhibiting MDM2, MA242 stabilized p53, leading to increased apoptosis and cell cycle arrest. Furthermore, since MDM2 could also regulate p21 independent of p53, the observed upregulation of p21 further supported this mechanism. Additionally, MA242 promoted apoptosis by increasing pro-apoptotic proteins such as cleaved PARP while simultaneously reducing the anti-apoptotic protein Bcl-2. This shift towards apoptosis, independent of p53 status, indicated that MA242 has the potential to overcome resistance mechanisms commonly faced in cancer therapy.

The therapeutic potential of MA242 was further confirmed in orthotopic breast cancer models, where its effectiveness closely matched the *in vitro* findings. In both the MCF7 and MDA-MB-231 models, MA242 significantly inhibited tumor growth without causing notable toxicity, emphasizing its favorable therapeutic index and safety profile. Our current data show that treated mice maintain stable body weights and exhibit no significant organ abnormalities, which supports the tolerability of MA242. However, we plan to improve our toxicity assessments in future studies by incorporating serum biochemistry analysis, hematological analysis, and long-term toxicity studies to evaluate liver and kidney function, blood parameters, and the chronic effects of treatment. In addition, the differential response observed in PDX models with varying MDM2 expression levels underscored the importance of MDM2 expression for the efficacy of MA242 treatment. Tumors with higher MDM2 expression responded significantly better, indicating that MDM2 is a critical factor in achieving therapeutic success. These results are consistent with our previous findings in pancreatic cancer (Wang et al., 2018) and HCC (Wang et al., 2019c) models, demonstrating that MA242's MDM2-targeting activity is consistent across various cancer types. This suggested MA242's potential as a broad-spectrum anticancer agent.

Cancer metabolism is characterized by altered metabolic pathways that support rapid cell proliferation and survival (Pavlova et al., 2022). TNBC cells exhibit significant metabolic reprogramming, including a pronounced glycolytic phenotype that converts glucose to lactate even in the presence of oxygen, supporting rapid proliferation and survival in the tumor microenvironment (Gandhi and Das, 2019). They have higher acidification and oxygen consumption rates than luminal breast cancer cells, driven by factors like EGF signaling and c-MYC, which suppress thioredoxin-interacting protein (TXNIP), an inhibitor of glycolysis (Sun et al., 2020; Wang Z. et al., 2020). TNBC also displays significant metabolic plasticity, heavily relying on fatty acid metabolism, including both synthesis and oxidation (Sun et al., 2020; Wang Z. et al., 2020). Fatty acid synthesis is upregulated in TNBC for membrane production and signaling, with fatty acid synthase (FASN) being overexpressed. Fatty acid oxidation (FAO), crucial for ATP production, supports TNBC proliferation and metastasis, with MYC-overexpressing tumors being sensitive to FAO inhibitors (Sun et al., 2020; Wang Z. et al., 2020). TNBC also acquires fatty acids from the blood or adipocytes via transporters like CD36 and Fatty acid-binding protein 5 (FABP5), with FABP5 loss reducing proliferation and invasion (Sun et al., 2020; Wang Z. et al., 2020). Additionally, alterations in amino acid metabolism are notable, with decreased glutamine but increased choline and glutamate levels, indicating a reliance on glutaminolysis and an active TCA cycle (Sun et al., 2020; Wang Z. et al., 2020). Elevated levels of succinate and isoleucine suggest enhanced TCA cycle activity (Sun et al., 2020; Wang Z. et al., 2020). The glutamine, serine, and glycine metabolic pathways are significantly upregulated, with key genes in these pathways overexpressed in TNBC cells (Sun et al., 2020; Wang Z. et al., 2020). Targeting these metabolic pathways offers potential therapeutic strategies for TNBC. Interestingly, MA242's impact on these pathways, as identified in this study, added another layer of understanding to its anticancer activity in breast cancer.

The downregulation of MDM2 by MA242 was particularly significant, given MDM2’s involvement in metabolic regulation. MDM2 is well-known for its role as a negative regulator of the p53 tumor suppressor (Oliner et al., 2016). However, its oncogenic influence extends beyond p53 regulation, impacting various metabolic pathways critical for cancer cell survival. Riscal et al. have reported that MDM2 is recruited to chromatin independently of p53, where it regulates amino acid metabolism and redox balance (Riscal et al., 2016). Specifically, MDM2’s interaction with ATF3/4 transcription factors appear crucial for tethering it to chromatin, thereby influencing genes involved in amino acid pathways (Riscal et al., 2016). In p53-deficient cells, depletion of MDM2 disrupts serine/glycine metabolism, the NAD^+^/NADH ratio, and glutathione (GSH) recycling, adversely affecting redox balance and tumor growth (Riscal et al., 2016). In liposarcoma (LPS) cells, treatment with Nutlin-3A (an MDM2-p53 interaction inhibitor) stabilizes p53 but also increases chromatin-bound MDM2, enhancing the expression of genes related to amino acid metabolism and promoting oncogenic activity, which may explain the poor clinical efficacy of these inhibitors (Cissé et al., 2020). Conversely, inhibiting chromatin-bound MDM2 with SP141, an MDM2 degrader discovered in our lab (Wang et al., 2014a; Wang et al., 2014b), promotes MDM2 degradation and disrupts *de novo* serine synthesis, thereby inhibiting LPS growth (Cissé et al., 2020). In our study, MA242 also disrupted amino acid metabolism and nicotinamide metabolism. This disruption mirrored the effects observed with MDM2 depletion, confirming MDM2’s regulatory role in these pathways. Changes in metabolites like L-aspartate, citrate, and oxaloacetate demonstrated MA242's broad impact on amino acid metabolism and its link to the TCA cycle and nucleotide synthesis. MA242 treatment decreased both NAD^+^ and NADH levels, although the NAD^+^/NADH ratio remained unchanged. This reduction suggested that MA242 affected mitochondrial function and glycolytic activity, essential for cellular energy production and redox balance. The stable NAD^+^/NADH ratio indicated that although the total levels of these cofactors were decreased, their relative balance was maintained, possibly to ensure continued metabolic function under stress conditions. In addition, MA242 also significantly disrupted GSH metabolism, resulting in an increased GSSG/GSH ratio and the induction of oxidative stress. This disruption aligned with MDM2’s role in regulating redox homeostasis and highlighted the therapeutic potential of targeting this pathway to induce oxidative stress in cancer cells. Elevated oxidative stress likely caused cellular damage and triggered apoptosis, which might reduce the tumorigenic potential of cancer cells.

MDM2 integrates respiration and mitochondrial bioenergetics independently of p53 (Arena et al., 2018; Rubio-Patiño et al., 2019). Cytosolic MDM2 translocates to the mitochondria, where it suppresses the transcription of NADH-dehydrogenase 6 (MT-ND6) in the mitochondrial genome, inhibiting respiration and inducing reactive oxygen species (ROS) production (Arena et al., 2018; Rubio-Patiño et al., 2019). This mitochondrial localization of MDM2 leads to ultrastructural changes, such as reduced matrix electron density and altered cristae, without increasing apoptosis (Arena et al., 2018; Rubio-Patiño et al., 2019). These morphological alterations indicate that MDM2 can profoundly impact mitochondrial structure and function, suggesting a role in modulating cellular energy production and stress responses (Arena et al., 2018; Rubio-Patiño et al., 2019). MA242’s impact on nucleotide metabolism is profound. The observed time-dependent increase in adenosine and guanosine nucleotides demonstrated the compound’s role in stimulating purine synthesis. This stimulation is indicative of a targeted intervention in purine metabolic pathways, which are essential for DNA and RNA synthesis and thus, crucial for rapidly proliferating cancer cells (De Vitto et al., 2021). Furthermore, MA242’s effects on pyrimidine metabolism reveal a nuanced alteration in nucleotide balance. The increase in UMP and CDP levels, alongside a decrease in UTP and CTP, suggests a disruption in *de novo* pyrimidine synthesis. The reduction in carbamoyl-aspartate, coupled with elevated aspartate levels, implies that MA242 impairs pyrimidine synthesis while simultaneously enhancing purine pathways. This dual action highlights MA242's specificity in modulating nucleotide metabolism rather than causing a broad metabolic disturbance.

Our study demonstrates that MA242 treatment significantly disrupts nucleotide metabolism, impairs nicotinamide metabolism, and induces oxidative stress by affecting redox balance in breast cancer cells. These pathways are well-documented as essential for breast cancer development, progression, and therapy resistance, with evidence supporting their critical role in sustaining cancer cell survival and proliferation (Wang L. et al., 2020; Zheng et al., 2022; Das et al., 2024). While these pathways are essential, their dominance in driving breast cancer metabolism may vary depending on cancer subtype, genetic mutations, and microenvironmental factors. In our study, the significant disruption of these pathways by MA242 highlights their importance in breast cancer biology and supports their relevance as therapeutic targets.

Overall, MDM2 is a crucial regulator of cancer metabolic programming, independent of its interaction with p53, highlighting its significance in maintaining the metabolic flexibility required for cancer cells’ rapid growth and survival. The novel MDM2 inhibitor MA242 effectively disrupts these metabolic pathways, illustrating the pivotal role of MDM2 in supporting cancer cell proliferation and survival. By targeting MDM2’s p53-independent metabolic functions, MA242 impedes cancer cell growth and significantly alters metabolic pathways. These findings emphasize the therapeutic potential of targeting MDM2 to exploit the metabolic vulnerabilities of cancer cells. Future research should clarify how MDM2 inhibitors, such as MA242, regulate metabolism, their effects on normal cell mitochondrial function, and their role in toxicity. Conducting mitochondrial function assays in both cancerous and normal cells will help illuminate the metabolic mechanisms underlying MA242's activity. By comparing the metabolic responses of cancerous and normal tissues, we aim to identify biomarkers of therapeutic response, assess potential toxicity, and enhance our understanding of MA242's mechanism of action. Additionally, studies should explore how inhibitors like MA242 can be integrated into comprehensive cancer treatment strategies, including combination therapies. This approach aims to enhance effectiveness, overcome resistance, minimize toxicity, and ultimately improve patient outcomes.

## Data Availability

The original contributions presented in the study are included in the article/supplementary material, further inquiries can be directed to the corresponding authors.
